# Busy day effect on the use of obstetrical interventions and epidural analgesia during labour: a cross-sectional register study of 601 247 deliveries

**DOI:** 10.1186/s12884-022-04798-6

**Published:** 2022-06-13

**Authors:** Riitta Vilkko, Sari Räisänen, Mika Gissler, Vedran Stefanovic, Ilkka Kalliala, Seppo Heinonen

**Affiliations:** 1 Faculty of Medicine, Doctoral Programme in Clinical Research, University of Helsinki, Haartmaninkatu 8, 00290 Helsinki, Finland; 2 Tampere University of Applied Sciences, Kuntokatu 3, 33520 Tampere, Finland; 3 Information Services Department, THL Finnish Institute for Health and Welfare, Mannerheimintie 166, 00270 Helsinki, Finland; 4 Department of Obstetrics and Gynecology, Fetomaternal Medical Center, Helsinki University Hospital and University of Helsinki, Haartmaninkatu 2, 00290 Helsinki, Finland; 5 Department of Obstetrics and Gynecology, Helsinki University Hospital and University of Helsinki, Haartmaninkatu 2, 00290 Helsinki, Finland; 6 Department of Metabolism, Digestion and Reproduction & Department of Surgery and Cancer, Faculty of Medicine, Imperial College London, London, UK

**Keywords:** Busy day effect, Delivery volume, Hospital size, Intervention during labour, Obstetrics, Obstetrical intervention, Register study

## Abstract

**Background:**

Daily delivery volume might affect the quality of obstetric care. We explored the busy day effect on selected obstetrical interventions and epidural analgesia performed during labour in different sized delivery hospitals and on the Finnish obstetric ecosystem.

**Methods:**

We conducted a cross-sectional study on Finnish Medical Birth Register data of singleton pregnancies (*N* = 601,247) from 26 delivery hospitals from 2006 to 2016. Delivery hospitals were stratified by annual delivery volume: C (category) 1: < 1000, C2: 1000–1999, C3: 2000–2999, C4: ≥3000, and C5: university hospitals. The exposure variables were defined as quiet, optimal, and busy days determined based on daily delivery volume distribution in each hospital category. Quiet and busy days included approximately 10% of the lowest and highest delivery volume days, while the rest were defined as optimal. Outcome measures were unplanned caesarean section (CS), instrumental delivery, induction of labour, and epidural analgesia. We compared the incidence of outcomes in quiet vs. optimal, busy vs. optimal, and busy vs. quiet days using logistic regression. The statistical significance level was set at 99% to reduce the likelihood of significant spurious findings.

**Results:**

In the total population, the incidence of instrumental delivery was 8% (99% CI 2–15%) lower on quiet than on optimal days. In smaller hospitals (C1 and C2), unplanned caesarean sections were performed up to one-third less frequently on busy than optimal and quiet days. More (27%, 99% CI 12–44%) instrumental deliveries were performed in higher delivery volume hospitals (C4) on busy than quiet days. In C1-C3, deliveries were induced (12–35%) less often and in C5 (37%, 99% CI 28–45%) more often on busy than optimal delivery days. More (59–61%) epidural analgesia was performed on busy than optimal and quiet days in C4 and 8% less in C2 hospitals.

**Conclusions:**

Pooled analysis showed that busyness had no effect on outcomes at the obstetric ecosystem level, but 10% fewer instrumental deliveries were performed in quiet than on busy days overall. Furthermore, dissecting the data shows that small hospitals perform less, and large non-tertiary hospitals perform more interventions during busy days.

## Background

Quality improvement efforts in obstetric care aim to ensure and improve safety during patient care [1]. However, errors may still be inevitable as humans conduct obstetric care [2]. Elective caesarean section (CS) is still the only delivery mode where it is possible to schedule the time of birth beforehand if the pregnancy proceeds to the date of surgery [3]. Otherwise, the unpredictable nature of natural birth causes challenges for delivery hospital organisations, especially when the variations of the daily patient flow may lead to relative under- and over-resourcing of staffing compared to the delivery hospital’s optimal capacity [4]. Busyness in health care is a widely recognised phenomenon, but the measurement tools and the scientific background of the natural causes of busy days are missing [5]. The delivery hospital-level ability to sustain standard care during busy days might be a potential quality indicator of obstetric care [6]. Extensive population-based data are needed to evaluate obstetric care quality based on hospital-level outcome measures [7–9].

Our earlier study showed that more blood transfusions were performed during labour during busy days, regardless of the size of the delivery hospital [10]. It is, therefore, possible that during busy days the delivery hospital’s capacity to offer other obstetrical interventions and epidural analgesia during labour might vary compared to optimal or quiet patient flow days. Here we aimed to explore the busy day effect on the incidence of obstetrical interventions and the use of epidural analgesia during labour in different sized delivery hospitals and on the obstetric ecosystem level.

## Methods

We performed a cross-sectional register study with data on singleton deliveries between 2006 and 2016 in Finland. The data were collected from the Finnish Medical Birth Register (MBR), which provides data on all live births and stillbirths with a birth weight of ≥500 g or gestational age of ≥22 weeks in Finland since 1987. MBR data contains comprehensive information on maternal sociodemographic profiles and pregnancy, delivery and postpartum characteristics, outcomes, and diagnoses defined by the International Classification of Diseases and Related Health Problems (ICD-10). Data on newborn characteristics, diagnoses, and outcomes cover the first seven postnatal days [11]. Finland is a Northern country with 5.5 million inhabitants where almost all (99%) women are using public prenatal care services that are free of charge. All delivery care services are publicly funded and operated at local, central, and university levels. From the perspective of patient safety, the quality of obstetric care in Finland is rated as one of the best globally [12]. The total study population included 634,810 singleton hospital deliveries from 34 delivery hospitals. Deliveries in eight hospitals closed during the study period due to very low annual delivery volume (3.8%, 24,414 of 634,810), and multiple pregnancies (1.4%, 9149 of 634,810) were excluded. After these exclusions, data for this study included 601 247 singleton deliveries that occurred in 26 delivery hospitals.

We categorized all 26 delivery hospitals into five categories based on their annual delivery volume and profile (C1-C5). Hospital categorisation was designed based on our recently published study, which showed that daily delivery distribution, and patient characteristics varied by delivery hospital annual delivery volume and profile [6]. In the present study, delivery hospital category C1 included local and central level delivery hospitals (*n* = 7) with low annual delivery volumes (< 1000 annual deliveries). Hospital categories C2 (*n* = 10) and C3 (*n* = 2) included local and central level delivery hospitals across the country, with larger annual delivery volumes from 1000 to 1999 and 2000 to 2999, respectively. Hospital category C4 (*n* = 2) included large-sized central level delivery hospitals (≥3000 annual deliveries) near the capital area. Hospital category C5 had five university hospitals with a profile of treating the most complicated cases. Some of the extreme high-risk pregnancies and all extreme pre-term deliveries are referred to Helsinki University Hospital. The referral system covers the whole country. Treating the university hospitals as one category enables more reliable analyses due to differences in patient risk profiles between university hospitals and other similar-sized delivery hospitals.

The exposure of the study was the daily delivery volume, defined as quiet, optimal, and busy. Daily delivery volume categorisation was performed separately for each five hospital categories. Approximately 10% of the lowest delivery volume days were quiet days, and about 10% of the highest delivery volume days were busy. Days between quiet and busy days were defined as optimal delivery volume days. In the total population, 10.2% (61,059 of 601,247) and 9.5% (57,419 of 601,247) were defined as quiet and busy days, respectively. Days between quiet and busy days (80.3%; 482,769 of 601,247) were optimal delivery volume days.

The outcome measures were selected based on the data availability and the earlier supporting literature associated with the commonness of performed obstetric interventions during obstetric care leading to possible hospital-level patient flow variations [13–18]. Outcome measures were selected as unplanned caesarean section (unplanned CS), instrumental delivery, induction of labour, and epidural analgesia (EA) to represent the delivery hospital’s capacity to perform obstetrical interventions and epidural analgesia during labour. Unplanned CS included all unplanned CSs, completed for maternal causes or signs of fetal compromise. Instrumental deliveries included all vacuum extraction assisted deliveries and forceps. The outcome measure of induced labour consists of all amniotomies and medical induction of labour by oxytocin or prostaglandin analogues. The data of balloon catheters used for the labour induction were not available, and for the lack of information was not included in the study. EA as an outcome measure consists of all EAs performed for pain relief during labour, excluding the number of EAs served for unplanned CSs.

We performed several bivariate and multivariate statistical analyses to study the busy day effect on the total population and separately on five hospital categories. All five hospital categories were pooled for the analyses of the total population-level data on daily delivery volume. Incidence of outcome measures was compared between quiet vs. optimal days (reference), busy vs. optimal days (reference), and busy vs. quiet days (reference). Multivariate analyses were performed using logistic regression analysis methods, and both crude and adjusted odds ratios (aORs) with a 99% statistical significance level were determined.

Previous studies and bivariable statistical (Chi-Square) tests selected maternal age, parity, and infant birth weight as confounding variables. For the analyses, maternal age was categorised as: < 25, 25 to 34, and ≥ 35 years. Parity was defined as nulliparous (no previous deliveries) or multiparous (one or more previous deliveries). The infant’s birth weight was categorised as < 3000 g, 3000-3999 g, and ≥ 4000 g. The pooled summary effect estimates across all five hospital categories with corresponding 99% confidence intervals for the total population were calculated based on the aORs using the inverse variance weighted random-effects method [19]. In addition, the heterogeneity between studies was assessed with the Cochran Q test and the I2 metric of inconsistency [20]. Analyses were conducted with SPSS statistical software (version 26) and STATA/SE 16 (StataCorp, College Station, TX, USA).

## Results

Table 1 reports data on delivery volume distribution (mean and range) in total population and the number of days and deliveries on quiet, optimal, and busy days in five hospital categories. Maternal and fetal demographics and characteristics in each hospital category are reported in Table 2.

**Table 1:** Hospital categorisation, daily delivery volume during the study period (n, %, min-max) and varying daily patient flow (n, %, min-max) by the size of delivery hospitals

Delivery hospital categorisation	Total delivery volume during the study period	Mean of daily deliveries	Range of daily deliveries	Range of daily deliveries by varying daily patient flow (min-max)			Daily delivery volume with varying daily patient flow (n, %)		
Delivery hospital category by annual delivery volume/profile	n (%)	n	min-max	Quiet days	Optimal days	Busy days	Quiet days	Optimal days	Busy days
C1: < 1000	55,448 (9.2)	2.0	1–10	1	2–5	6–10	7212 (13.0)	44,056 (79.5)	4180 (7.5)
C2: 1000–1999	165,573 (27.5)	4.6	1–16	1–2	3–8	9–16	14,200 (8.6)	136,711 (82.6)	14,662 (8.9)
C3: 2000–2999	54,574 (9.1)	4.5	1–19	1–4	5–11	12–24	5303 (9.7)	42,698 (78.2)	6573 (12.0)
C4: ≥3000	108,254 (18.0)	13.5	1–34	1–8	9–23	24–34	9731 (9.0)	88,156 (81.4)	10,367 (9.6)
C5: University hospitals	217,398 (36.2)	10.8	1–30	1–7	8–18	19–30	24,613 (11.3)	171,148 (78.7)	21,637 (10.0)
Total	601,247 (100.0)	7.1	1–34	1–8	2–23	6–34	61,059 (10.2)	482,769 (80.3)	57,419 (9.5)

Quiet days = the number of deliveries that occurred during the closest 10% of the lowest daily delivery volume days

Optimal days = the number of deliveries occurred between the lowest (10%) and highest (10%) delivery volume days

Busy days = the number of deliveries that occurred during the closest 10% of the highest daily delivery volume days

C = category

**Table 2:** Maternal and fetal characteristics, selected outcome measures by delivery hospital category and daily delivery volume categorisation

Delivery hospital category	Maternal and fetal characteristics and studied outcomes	Categorisation	Quiet day	Optimal day	Busy day
C1	Maternal age (n, %)	< 25	1438 (19.9)	9225 (20.9)	886 (21.2)
		25–34	4465 (61.9)	27,039 (61.4)	2522 (60.3)
		≥35	1309 (18.2)	7792 (17.7)	772 (18.5)
	Parity (n, %)	Nulliparous	2788 (38.7)	16,339 (37.1)	1513 (36.2)
	Birthweight (n, %)	< 3000	909 (12.6)	5329 (12.1)	475 (11.4)
		3000–3999	5078 (70.4)	31,126 (70.7)	2952 (70.6)
		≥4000	1224 (17.0)	7586 (17.2)	753 (18.0)
	Outcome (n, %)	Unplanned CS***	748 (10.4)	3953 (9.0)	311 (7.4)
		Instrumental delivery	524 (7.3)	3317 (7.5)	294 (7.0)
		Induction***	1608 (22.3)	9254 (21.0)	783 (18.7)
		EA	2478 (34.4)	15,398 (35.0)	1381 (33.0)
C2	Maternal age (n, %)	< 25	2732 (19.2)	26,565 (19.4)	2815 (19.2)
		25–34	8938 (62.9)	86,463 (63.2)	9259 (63.1)
		≥35	2530 (17.8)	23,683 (17.3)	2588 (17.7)
	Parity (n, %)	Nulliparous	5705 (40.2)	54,747 (40.0)	5781 (39.4)
	Birthweight (n, %)	< 3000	1936 (13.6)	17,695 (12.9)	1913 (13.1)
		3000–3999	9854 (69.4)	95,316 (69.7)	10,120 (69.0)
		≥4000	2408 (17.0)	23,678 (17.3)	2625 (17.9)
	Outcome (n, %)	Unplanned CS***	1415 (10.0)	12,440 (9.1)	1267 (8.6)
		Instrumental delivery	1237 (8.7)	11,984 (8.8)	1197 (8.2)
		Induction***	2632 (18.5)	25,100 (18.4)	2437 (16.6)
		EA***	5659 (39.9)	54,711 (40.0)	5580 (38.1)
C3	Maternal age (n, %)	< 25	1092 (20.6)	8310 (19.5)	1248 (19.0)
		25–34	3316 (62.5)	26,870 (62.9)	4184 (63.7)
		≥35	895 (16.9)	7518 (17.6)	1141 (17.4)
	Parity (n, %)	Nulliparous ***	2044 (38.5)	16,485 (38.6)	2583 (39.3)
	Birthweight (n, %)	< 3000 ***	585 (11.0)	5110 (12.0)	863 (13.1)
		3000–3999***	3636 (68.6)	29,468 (69.1)	4569 (69.6)
		≥4000***	1082 (20.4)	8096 (19.0)	1135 (17.3)
	Outcome (n, %)	Unplanned CS	561 (10.6)	4563 (10.7)	683 (10.4)
		Instrumental delivery	433 (8.2)	3743 (8.8)	556 (8.5)
		Induction***	1522 (28.7)	10,329 (24.2)	1125 (17.1)
		EA	1631 (30.8)	13,636 (31.9)	2083 (31.7)
C4	Maternal age (n, %)	< 25***	1182 (12.1)	11,331 (12.9)	1227 (11.8)
		25–34***	6428 (66.1)	57,858 (65.6)	6668 (64.3)
		≥35***	2121 (21.8)	18,967 (21.5)	2472 (23.8)
	Parity (n, %)	Nulliparous***	4103 (42.2)	41,445 (47.0)	5115 (49.3)
	Birthweight (n, %)	< 3000***	1071 (11.0)	10,793 (12.3)	1401 (13.5)
		3000–3999***	6924 (71.4)	62,362 (70.9)	7371 (71.2)
		≥4000***	1706 (17.6)	14,744 (16.8)	1587 (15.3)
	Outcome (n, %)	Unplanned CS***	835 (8.6)	8410 (9.5)	1084 (10.5)
		Instrumental delivery ***	796 (8.2)	8827 (10.0)	1155 (11.1)
		Induction***	1698 (17.4)	17,201 (19.5)	2604 (21.1)
		EA***	4236 (43.5)	38,923 (44.2)	5760 (55.6)
C5	Maternal age (n, %)	< 25***	4530 (18.4)	27,935 (16.3)	3138 (14.5)
		25–34***	15,168 (61.1)	108,552 (63.4)	13,879 (64.1)
		≥35***	4915 (20.0)	34,661 (20.3)	4620 (21.4)
	Parity (n, %)	Nulliparous***	10,328 (42.0)	72,826 (42.6)	9620 (44.5)
	Birthweight (n, %)	< 3000***	4017 (16.3)	26,115 (15.3)	3334 (15.4)
		3000–3999***	16,694 (67.8)	115,890 (67.7)	14,708 (68.1)
		≥4000***	3895 (15.8)	29,051 (17.0)	3565 (16.5)
	Outcome (n, %)	Unplanned CS	2499 (10.2)	17,240 (10.1)	2277 (10.5)
		Instrumental delivery***	1865 (7.6)	14,560 (8.5)	1871 (8.6)
		Induction	5091 (20.7)	34,106 (19.9)	4241 (19.6)
		EA***	11,736 (47.7)	80,655 (47.1)	10,484 (48.5)

C1 = Delivery hospitals with < 1000 annual deliveries

C2 = Delivery hospitals with 1000–1999 annual deliveries

C3 = Delivery hospitals with 2000–2999 annual deliveries

C4 = Delivery hospitals with ≥3000 annual deliveries

C5 = University hospitals

Quiet days = the number of deliveries that occurred during the closest 10% of the lowest daily delivery volume days

Optimal days = the number of deliveries occurred between the lowest (10%) and highest (10%) delivery volume days

Busy days = the number of deliveries that occurred during the closest 10% of the highest daily delivery volume days

*** *p* < .001

**Fig. 1:**
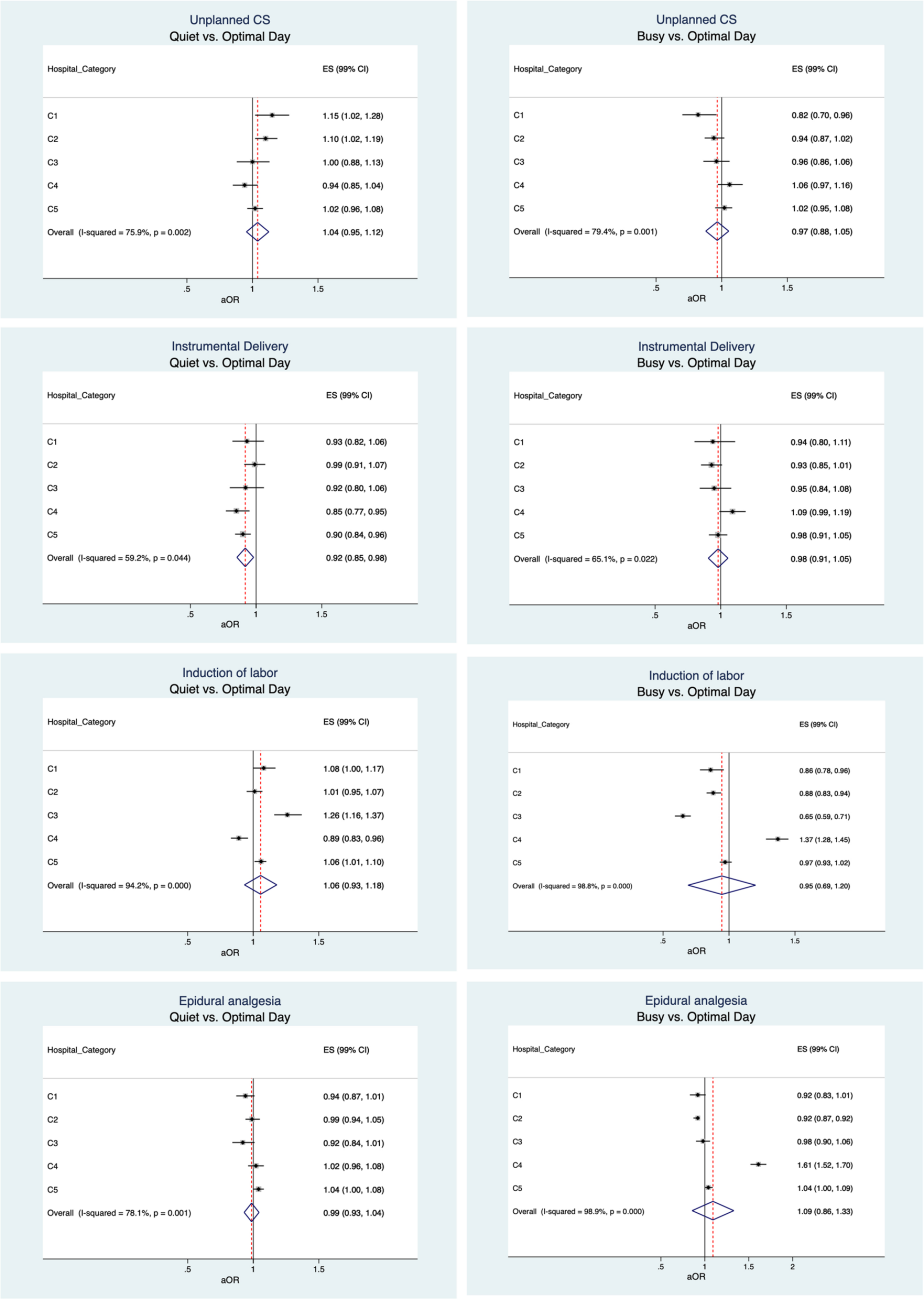
Adjusted OR (aOR) and 99% confidence intervals (CIs) of unplanned SC, instrumental delivery, induction of labour and epidural analgesia categorised by daily delivery volume (quiet vs. optimal day and busy vs. optimal day) in five delivery hospital categories. C1 = Delivery hospitals with < 1000 annual deliveries. C2 = Delivery hospitals with 1000–1999 annual deliveries C3 = Delivery hospitals with 2000–2999 annual deliveries. C4 = Delivery hospitals with ≥3000 annual deliveries. C5 = University hospitals. Unplanned CS=Unplanned caesarean section. EA = Epidural analgesia. Quiet days = the number of deliveries that occurred during the closest 10% of the lowest daily delivery volume days. Optimal days = the number of deliveries occurred between the lowest (10%) and highest (10%) delivery volume days. Busy days = the number of deliveries that occurred during the closest 10% of the highest daily delivery volume days. aOR adjusted by maternal age (categorical), parity (nulliparous or multiparous), birthweight (categorical). ** p < 0.05. *** p < .001

**Fig. 2:**
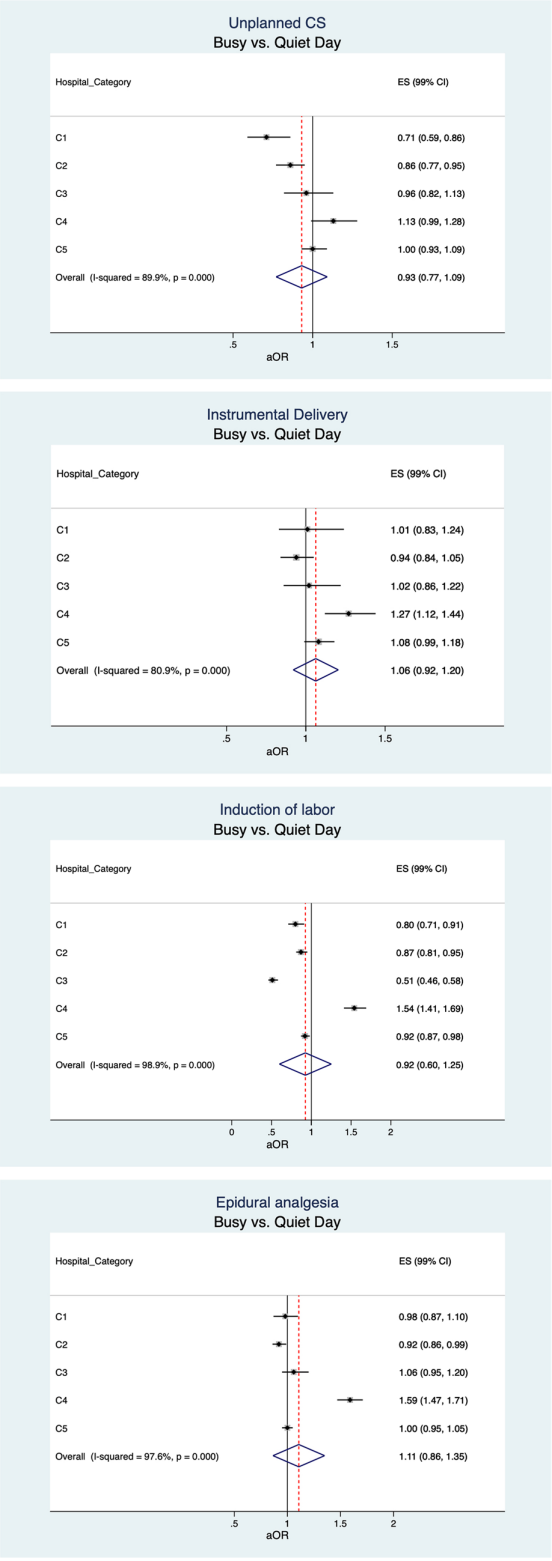
Adjusted OR (aOR) and 99% confidence intervals (CIs) of unplanned SC, instrumental delivery, induction of labour and epidural analgesia categorised by daily delivery volume (quiet vs. busy day) in five delivery hospital categories. C1 = Delivery hospitals with < 1000 annual deliveries. C2 = Delivery hospitals with 1000–1999 annual deliveries C3 = Delivery hospitals with 2000–2999 annual deliveries. C4 = Delivery hospitals with ≥3000 annual deliveries. C5 = University hospitals. Unplanned CS=Unplanned caesarean section. EA = Epidural analgesia. Quiet days = the number of deliveries that occurred during the closest 10% of the lowest daily delivery volume days. Optimal days = the number of deliveries occurred between the lowest (10%) and highest (10%) delivery volume days. Busy days = the number of deliveries that occurred during the closest 10% of the highest daily delivery volume days. aOR adjusted by maternal age (categorical), parity (nulliparous or multiparous), birth weight (categorical). ** p < 0.05. *** p < .001

At the population level, the only statistically significant difference was that instrumental deliveries were performed 8% (99% CI 2–15%) less on quiet than optimal delivery volume days (Figs. 1 and 2).

The average annual delivery volume by delivery hospital’s size ranged from 720 to 4921 in the hospital categories C1-C4 and from 2409 to 5630 in the university hospitals during the 11-year study period. Unplanned caesarean sections were performed 15% (99% CI 2–28%) and 10% (99% CI 2–19%) more in C1 and C2, respectively, on quiet than optimal days and 18% (99% CI 4–13%) and 29% (99% CI 14–41%) less on busy than optimal and quiet days, respectively in hospital category C1 (Figs. 1 and 2). Instrumental deliveries were performed 10% (99% CI 4–16%) and 15% (99% CI 5–23%) less on quiet than optimal days in C5 and C4, respectively, and 27% (99% CI 12–44%) more on busy than quiet days in C4 (Figs. 1 and 2). Labour induction was performed 6% (99% CI 1–10%) and 26% (99% CI 16–37%) more on quiet days in C5 and C3, respectively, and 11% (99% CI 4–17%) less in hospital category C4 than on optimal days (Fig. 1). On busy days, deliveries were induced 12–49% less in C1-C3 and 37–54% more in C4 than on optimal and quiet days (Figs. 1 and 2). Epidural analgesia was used 8% less in C2 and 59–61% more in C4 on busy than optimal and quiet days (Figs. 1 and 2).

## Discussion

We studied busy day effects on obstetric care using daily delivery volume by hospital size category stratified into quiet, optimal, and busy days as the primary exposure. The outcome measures were unplanned CS, instrumental delivery, induction of labour, and EA during labour in different sized delivery hospitals and on the whole obstetric ecosystem level. The only significant difference in pooled analysis comprising the entire delivery hospital network was the less than 10% decrease of instrumental deliveries on quiet compared to optimal days.

Dissecting the data by hospital size brought up at least four interesting findings. First, the unplanned CS rate decrease was 15 to 30% from quiet to busy days in the smallest hospitals (C2, C1), whereas there was no change in the other hospital categories in the same comparison. Second, in C3 hospitals, induction showed a similar decreasing pattern as CSs from quiet to busy days. Still interestingly, in large non-university hospitals (C4), the induction rate showed the opposite and increased significantly up to 50% from quiet to busy days. Third, epidural analgesia was up to 60% higher on busy than quiet days in large non-university hospitals (C4) but not in other hospital categories. Fourth, adjustment for the case mix changed the results only a little, suggesting that the patient profiles in each hospital category on quiet and busy days were very similar.

In small delivery hospitals (C1, C2), unplanned CS rates were higher during quiet days and lowered during busy days. This cannot be explained by medical reasons or case-mix differences since these were considered in the multiple regression and in the study setting, where each hospital category served as its control. Therefore, there is no reason to think that the treatment policy is deliberately changed by daily variation. Unplanned CS is the most intensive intervention in resource use since it takes more time than instrumental delivery, and EA and a surgical team are needed [21]. Therefore, we can speculate that the capacity to perform unplanned CS does not fully cover the demand on busy days in small delivery hospitals. This is in line with previous research results showing that busy times like night and weekend deliveries are associated with unfavourable alterations in some obstetric interventions and perinatal outcomes [22–24].

In general, large non-university hospitals (C4) performed more unplanned CSs, instrumental deliveries, inductions, and EAs on busy compared to quiet days. This result was somewhat unexpected and not noticed in other delivery hospital categories. However, it shows that large central hospitals have significant capacity to perform and increase interventions even on busy days. The explanation why this happens is unclear. There may be an attempt to speed up the labour process with the increasing patient flow. Whatever the explanation, it is evident that inductions were organized in a very poorly coordinated way since more inductions were performed on busy than optimal or quiet days.

After pooling all hospital categories, the sum of available data showed that a 10% decrease in instrumental deliveries in quiet compared to optimal days was the only statistically significant finding over the full spectrum of outcomes at the ecosystem level. Still, the observed heterogeneity was high in all pooled analyses suggesting marked differences between hospital categories.

Differences between delivery hospital categories were significant in each outcome, which supports heterogeneity in delivery hospital comparison. In tertiary university hospitals (C5), the changes in the use of all interventions were less than 10% during busy and quiet days compared to optimal days and primarily did not reach nominal statistical significance, suggesting that the university hospitals were resistant to changes in patient flow.

The strength of this study is extensive, population-based national data from reliable MBR of high quality and comprehensive over a lengthy period [25, 26]. Such big data sets are needed to discover small but clinically significant differences caused by daily delivery volume variation, which would easily go unnoticed if only data from one delivery hospital were analysed for self-validation purposes. Furthermore, analyses were performed with a multivariate regression model, and the case-mix adjustment was performed for every outcome.

The quiet, optimal, and busy days were determined in different sized delivery hospitals. These daily delivery volume variation calculations were based on the daily delivery frequency in different sized hospital categories and estimated to the nearest 10% to represent quiet and busy delivery volume days. However, due to the complexity, duration, and nature of the delivery process, the calculations may not be exact, and the daily delivery volume does not describe the actual workload in the hospital during varying daily periods. The other limitation of this study is the hospital categorisation by annual delivery volume. The register data related lacks specific characteristics between different delivery hospitals, such as acuity, patient case-mix, availability of obstetric anesthesia, and detailed information on nursing staff in each unit.

## Conclusions

This study demonstrates that a large delivery hospital’s overall capacity to perform interventions during labour is generally good despite varying daily delivery volumes and busyness. In contrast, small delivery hospitals may have difficulties maintaining this ability. This is reflected by the unplanned CS rates being significantly higher in C1 and C2 hospitals on quiet than busy days. On the other hand, especially large non-university hospitals (C4) appeared to work in a very poorly coordinated way since they tended to induce more on busy than on optimal or quiet days. This, in turn, results in significant overuse of epidural analgesia. We, therefore, suggest that such quality assessment of the busy day effect based on outcome measures should be part of the quality improvement at delivery hospitals to ensure that treatment standards are met despite variations in daily patient flow.

## Data Availability

The data that support the findings of this study are available from the Finnish Institute of Health and Welfare. Still, restrictions apply to the availability of these data, which were used under license for the current study, are not publicly available. Data are, however, available from the authors upon reasonable request and with permission of the Finnish Institute of Health and Welfare.

## Abbreviations

aOR: Adjusted Odds ratio
cOR: Crude Odds Ratio
CI: Confidence interval
ICD −10: Classification of Diseases and Related Health Problems
EA: Epidural analgesia
MBR: Medical Birth Register
Unplanned CS: Unplanned caesarean section
